# A short tool to screen HIV-infected patients for mild neurocognitive disorders – a pilot study

**DOI:** 10.1186/2050-7283-2-21

**Published:** 2014-07-18

**Authors:** Dominique Fasel, Ursula Kunze, Luigia Elzi, Vreni Werder, Susanne Niepmann, Andreas U Monsch, Rahel Schumacher, Manuel Battegay

**Affiliations:** Division of Infectious Diseases & Hospital Epidemiology, University Hospital Basel, Petersgraben 4, Basel, 4031 Switzerland; Memory Clinic, Department of Geriatrics, University Hospital, Basel, Switzerland

## Abstract

**Background:**

We aimed to evaluate the accuracy and acceptability of a short screening test battery for mild neurocognitive deficits.

**Methods:**

HIV-infected individuals with a suppressed viral load were examined at the University Hospital Basel with a screening test consisting of a questionnaire and selected cognitive tests, administered by trained nurses, followed by an in-depth neuropsychological examination. Test acceptance was evaluated with a questionnaire.

**Results:**

30 patients were included in this study (median age of 52.5 years (interquartile range (IQR) 47–64), prior AIDS-defining condition in 37%, median CD4 cell count 658 (IQR 497–814) cells/μl). Overall, 25 (83%) patients were diagnosed with HIV-associated neurocognitive disorders (HAND) on in-depth neuropsychological assessment (16 patients had asymptomatic neurocognitive impairment (ANI), 8 a mild neurocognitive disorder (MND) and 1 patient HIV-associated dementia (HAD). Among 25 patients with HAND, only 9 patients (36%) were complaining of memory loss. The screening battery revealed neurocognitive deficits in 17 (57%) patients (sensitivity 64%, specificity 80%, positive predictive value 94% and negative predictive value 31%). Most patients (83%) estimated the screening test as valuable and not worrisome.

**Conclusions:**

A questionnaire combined with selected neuropsychological tests is a short, easy-to-perform very well accepted screening tool for mild neurocognitive disorders in asymptomatic HIV-infected individuals.

## Background

Combined antiretroviral therapy (cART) has dramatically changed the prognosis of HIV-infection (Mocroft et al. 2003; Weber et al. 2012; Stöckle et al. 2012; Jaggy et al. 2003; Egger et al. 2002). Given a timely diagnosis and treatment, life expectancy is most likely only marginally decreased compared to the general population (The Antiretroviral Therapy Cohort Collaboration 2008). Therefore, with increasing age of HIV patients, long-term aspects such as neurotoxic effects of the virus and possibly of treatments gain importance (Robertson et al. 2009). Losses in memory function, psychomotor speed and/or executive functions may occur at a higher frequency in HIV-infected compared to HIV-negative individuals (Robertson Robertson et al. 2009). Cognitive disorders may negatively impact behaviour (Hinkin et al. 2002), autonomy in everyday life, and risk behaviour (Gonzalez et al. 2005; Vance & Struzick 2007), leading to a diminished quality of life, lower adherence to cART and increased mortality. An early diagnosis of cognitive impairment is important for the initiation of cART which can then lead to improvements of neurocognitive symptoms (Cysique & Brew 2009; Joska et al. 2010; Tozzi et al. 2007).

Definition of HIV-associated neurocognitive disorders (HAND) include three conditions: asymptomatic neurocognitive impairment (ANI), HIV-associated mild neurocognitive disorder (MND) and HIV-associated dementia (HAD). The prevalence of HAND was estimated to be 69% in HIV-infected persons in Switzerland who have been successfully treated for many years (Simioni et al. 2010). In a US study (Robertson et al. 2007), 21% of asymptomatic HIV-infected individuals fulfilled the criteria for ANI. Subjective reports about cognitive symptoms seem to be unreliable as up to 64% of asymptomatic patients were found to have cognitive impairment on neuropsychological assessment (Simioni et al. 2010). A patient’s underestimation of his own cognitive deficits is possibly due to a deficit in meta-memory, i.e. an executive dysfunction (Woods et al. 2009). On the other hand, overestimation of one's own cognitive deficit is frequently seen in patients with depressive disorders (Rourke et al. 1999; Carter et al. 2003). Various screening tests like the HIV dementia scale (HDS) (Power et al. 1995), the EXIT interview (Berghuis et al. 1999), the Mental Alternation Test (Jones et al. 1993), the modified Memorial Sloan-Kettering Scale (Marder et al. 2003) or the International HIV Dementia Scale (IHDS) (Sacktor et al. 2005) are used to identify HIV associated dementia, but these tests are not sensitive enough to detect the milder forms of HAND, i.e. ANI and MND, which are more prevalent in the HIV population (Singh et al. 2010; Carey et al. 2004). Recently, a score ≤ 14 points on the HDS (Power et al. 1995) was found to yield a positive predictive value of HAND of 92% in complainers and 82% in non-complainers (Robertson et al. 2007).

A useful screening test must have acceptable psychometric properties. Carey et al. (Carey et al. 2004) were able to show that a combination of only two validated and standardised neuropsychological tests was better at classifying patients with cognitive disorders than the HDS alone. The neuropathological changes caused by the HIV infection mainly affect the fronto-striato-thalamo-cortical circuit, deficits in processing speed, executive functions and verbal episodic memory (Robertson et al. 2009; Woods et al. 2009). The most frequently used tests which are viewed as sensitive are the verbal memory tasks (Singh et al. 2010; Carey et al. 2004; Skinner et al. 2009), the Trail Making Test part A and B (1944; Tombaugh et al. 1998), the Grooved Pegboard Test (Ruff & Parker 1993), the Digit Symbol Test (Härting et al. 2000; Aster et al. 2006), and the Digit Span forwards and backwards (Härting et al. 2000). Combination of the Hopkins Verbal Learning Test – Revised (HVLT-R) (Benedict et al. 1998) with the Digit Symbol Test (Härting et al. 2000; Aster et al. 2006) or with the Grooved Pegboard Test (Ruff & Parker 1993) non-dominant hand yielded a sensitivity of 75-78% and a specificity of 85-92%, respectively, in detecting mild cognitive disorders in HIV-infected individuals (Carey et al. 2004).

Taking the above mentioned findings into account, the aims of this study were to evaluate the performance and to assess the acceptability of a German-language screening battery consisting of a short questionnaire and seven brief neuropsychological tests administered by trained nurses to screen for neurocognitive deficits in treated HIV-infected patients.

## Methods

### Ethical approval

The protocol was approved by the local Ethics Committee “Ethikkommission beider Basel”. All patients gave written informed consent.

### Study participants

Study participants were 30 HIV-infected individuals in care at the HIV Clinic of the University Hospital Basel, Switzerland meeting the following inclusion criteria: age ≥18 years, cART since ≥6 months, an undetectable HIV viral load (<50 copies/mL) for ≥3 months, and to be a German native speaker. Exclusion criteria were auditory, visual or motor deficits, clinical signs of disorientation, current injecting drug use, current major depression according to Diagnostic and Statistical Manual of Mental Disorders (Trull et al. 2012), neurologic or severe psychiatric conditions that affect cognition, and a history of opportunistic infection of the central nervous system within the last 2 years. The following data were collected at the time of the screening test and obtained from the prospective data collection of the Swiss HIV Cohort Study: age, education, gender, CDC stage, CD4 cell count, HIV viral load, co-infection with hepatitis C, co-medication, drug and alcohol consumption, history for cART, opportunistic diseases and syphilis. Medical history of thyroid or vitamin B12 deficiency was not reviewed.

### Study procedures and examination tools

Two study nurses were trained by a neuropsychologist on how to perform the screening battery according to standard procedures. The screening test consisted of a short questionnaire and seven selected neuropsychological tests based on theory-led principles and psychometric criteria, and it has already proven its value in a similar form in HIV-infected individuals (Carey et al. 2004). The time needed to perform the short examination was recorded and its acceptance was evaluated by a feedback questionnaire for both patients and nurses.

### Screening battery

Our screening battery comprised a questionnaire and a short examination of selective cognitive functions.

#### 1. Questionnaire

Following questions were asked to investigate cognitive functions: Do you frequently experience memory loss (e.g. do you forget the occurrence of special events even the more recent ones, appointments, etc.)? Do you feel that you are slower when reasoning, planning activities, or solving problems? Do you have difficulties in paying attention (e.g. to a conversation, a book, or a movie)? Patients could answer with ‘never’ , ‘rarely’ , ‘sometimes’ , ‘often’ or ‘always’.

As individuals may overestimate or underestimate their own deficits when making subjective statements on cognitive losses (Hinkin et al. 2002), we added two questions to increase the robustness of the subjective statements: one on everyday memory complaint because memory losses are frequently reported in this area (Woods et al. 2009): Do you intend to do something and then you forget what it was (e.g. do you go into another room to fetch something and then forget what you wanted to get)? The second refers to whether friends or family made remarks on the individual’s diminished cognitive skills: Do friends and/or members of your family tell you that your brain power has deteriorated?

The following two questions were asked to estimate whether there was a clinically relevant depression (Sacktor et al. 2005): How often did you note little interest or pleasure in doing things over the past 2 weeks? How often did you experience feeling down, depressed or hopeless over the past 2 weeks? Patients could answer with ‘not at all’ , ‘several days’ , ‘more than half the days’ or ‘nearly every day’.

#### 2. Examination of selected cognitive functions

Examination of selected cognitive functions consisted of seven brief tests to evaluate the following four domains: cognitive speed, memory, executive functions, and motor speed (Table 1).

**Table 1:** **List of the seven tests used to evaluate the four domains (cognitive speed, memory, executive function and motor speed)**

Cognitive domain	Test	Variables
Cognitive speed	Trail Making Test (TMT) (1944; Tombaugh et al. 1998) part A and B	number of seconds to complete part A and part B
	Digit Symbol Test (DST) (Härting et al. 2000; Aster et al. 2006)	number of correct items
Memory	wordlist from the Multiple Sclerose Inventarium Cognition (Calabrese et al. 2004)	number of correct items on 10 items learning and delayed recall
Executive functions	TMT ( 1944; Tombaugh et al. 1998) part A and B	number of seconds to complete part B
Motor speed	DST (Härting et al. 2000; Aster et al. 2006)	number of correct items
	Grooved Pegboard (Ruff & Parker 1993) with dominant and non-dominant hand	number of seconds needed for completion
	TMT ( 1944; Tombaugh et al. 1998) part A and B	number of seconds to complete part A and part B

Following the above some tests counted for more than one domain, e.g., if the result in TMT part A was below 1 standard deviation, it counted in the domains "cognitive speed" and "motor speed".

The domains were considered as pathological, if one result in this domain was pathological, ie, a standard score below -1.0. The cognitive screening was considered pathological if the patient had deficits in two or more domains.

Nurses who administered the screening test were provided with a table indicating pathological performance. For example, a TMT part A result of more than 40 seconds from a subject aged between 40 and 49 years was considered pathological.

### Acceptance of the screening battery

A feedback questionnaire was filled out by each patient and the study nurse to evaluate the acceptance of the screening test. The questionnaire for patients comprised the following questions: Is the test too difficult? Are the instructions clear? Does the test respect your privacy? Is the screening reasonable? Is the test burdening? Are you interested in the results of the examination? Is the test too long? The questionnaire for study nurses included following questions: Is the test too difficult for patients? Is the screening reasonable? Is the test burdening for the study nurse? Is the test too long?

The patients and nurses could answer on a scale of 1–5 (not at all – totally).

The questionnaire for nurses comprised also the following questions: Were there any ambiguities or uncertainties in the instructions? Were there any ambiguities or uncertainties in the evaluation? Were there any ambiguities or uncertainties in the interpretation?

The nurses were also able to attach comments or suggestions.

### In-depth neuropsychological assessment

Within one month, study participants were examined at the Basel Memory Clinic by a neuropsychologist using a comprehensive test battery to evaluate HAND. The examining neuropsychologist had no access to the results of the screening test.

The comprehensive neuropsychological examination, lasting for two hours, covered the following tasks: German version of the California Verbal Learning Test (Delis et al. 1987) (when age ≥ 50 years) or the Verbal Learning and Memory Test (Helmstadter et al. 2001) (when age < 50 years); Figural Fluency (Regard et al. 1982), modified Wisconsin Card Sorting Test (Nelson 1976); Rey-Osterrieth Complex Figure (Rey 1941); verbal fluency (semantic and phonemic) (Morris et al. 1989), Color Trails 1 and 2 (D’Elia et al. 1996); Boston Naming Test, 15 items (Nelson 1976); Digit Span (Härting et al. 2000; Aster et al. 2006), Color Word Interference Test (Stroop 1935) and Test of Attentional Performance (divided attention and alertness) (Fimm & Zimmermann 2009).

### Statistical analysis

Basic socio-demographic characteristics, CD4 cell count, and cART were compared using the Chi-square test or Fisher’s exact test for categorical variables, and the Mann–Whitney test for continuous variables. All analyses were performed using STATA software version 11 for Windows (STATA Corp, College Station, Texas, USA).

## Results

A total of 30 patients were included in this study between January 2011 and July 2011 at the HIV-Clinic of the University Hospital Basel. The median age was 52.5 years (interquartile range (IQR) 47–64) and most patients (87%) were males. One patient had a HIV viral load of 58 copies/mL, another one 65 copies/mL. Five patients had an elevated HIV viral load (range 86–3594 copies/mL) within 6 months before this investigation. The median CD4 cell count was 658 cells/μL (IQR 497–814); 11 patients (37%) had previously been diagnosed with an AIDS-defining infection, one of these suffering from cerebral toxoplasmosis 12 years before without clinically obvious neurological sequelae. Among co-morbidities, 3 patients (10%) had co-infection with hepatitis C, one patient had a history of transient ischemic attack many years before, and 5 patients (16.7%) had previously been treated for syphilis (stage I-II). Lumbar puncture yielding a negative syphilis serology of CSF was only done in one patient. We did not routinely carry out a lumbar puncture when patients had no clinical signs of involvement of the central nervous system between 6 months and 9 years before this investigation.

### Prevalence of HAND

Overall, 25 (83%) patients were diagnosed with HAND based on in-depth neuropsychological assessments. Of these, 16 patients (64%) had ANI, 8 (32%) MND, and 1 patient HAD. Among the 25 patients with HAND, only 9 patients (36%) were complaining of memory loss or difficulties to concentrate. The patient with HAD had HIV-infection CDC B3 with no relevant co-morbidity, in particular no obvious neurological disease. He was treated with an efavirenz-containing antiretroviral regimen. One of the 8 patients (13%) with MND and 5 (31%) of the 16 patients with ANI were also treated with an efavirenz-containing regimen. The 5 patients with a treated syphilis were diagnosed with ANI (n = 3), MND (n = 1), and no cognitive impairment (n = 1).

ANI was also diagnosed in one patient with stroke and in another patient with a history of cerebral toxoplasmosis. Also, two patients with occasional drug consumption (inhalative cocain, ketamin, methadon) had ANI. One patient with daily cannabis consumption had MND.

Detailed results of the in-depth neuropsychological examination are shown in Table 2.

**Table 2:** **Raw scores of the in-depth neuropsychological assessment**

	Max	IQR 25%	Median	IQR 75%	Range
California Verbal Learning Test (n = 18):					
Learning (trial 1–5)	80	43	47	54.75	26-63
Short Delay Free Recall	16	7.3	9.5	12.0	1-16
Long Delay Free Recall	16	7.3	11.0	12.0	1-16
Recognition Discriminability (%)	100%	90.9	93.2	95.5	81.2-95.5
Verbal Learning and Memory Test (n = 12):					
Learning (trial 1–5)	75	34.8	45.5	51.8	28-66
Delayed Recall	15	7	10	11	2-14
Figural Fluency	n.a.	25.3	32.0	36.5	8-57
Modified Wisconsin Card Sorting Test:					
Categories	6	6	6	6	1-6
Perseverative Errors	n.a.	0.0	0.5	3.0	0-8
Rey-Osterrieth Complex Figure:					
Copy	36	29.6	31.8	32.9	18.5-35.0
Immediate Recall	36	12.1	19.0	24.5	2.0-28.5
Delayed Recall	36	10.8	18.5	24.4	3.0-26.5
Semantic Fluency (Animals), 1’	n.a.	16.5	21	24	12-33
Phonemic Fluency (S-Words), 1’	n.a.	9	12	16	6-19
Color Trails 1	240“	33	37	49.75	19-126
Color Trails 2	240“	0	0	1	0-6
Boston Naming Test	15	14	15	15	11-15
Digit Span forward	12	6	7	8	4-11
Digit Span backward	12	5	5.5	6	2-10
Color Word Interference Test Time 1 (s)	n.a.	12	13	15	10-22
Color Word Interference Test Time 3/1	n.a.	1.85	2.17	2.40	1.53-3.76
Test of Attention Performance:					
Divided Attention Auditive (ms), Median	n.a.	516	555	631	329-1109
Divided Attention Visual (ms), Median	n.a.	758	830	898	599-1234
Alertness (ms), Median	n.a.	221	237	286	186-634

### Validity of questions addressing subjective cognitive impairment (SCI)

Twenty-five of the 30 patients were diagnosed with HAND based on the in-depth neuropsychological assessment. Among those, nine had reported a SCI (ie, sensitivity of SCI = 36%). Five patients had received a diagnosis of normal cognition after the in-depth neuropsychological assessment. Two of those had not reported SCI (ie, specificity of SCI = 40%). Thus, questions addressing SCI did not separate between those with and those without HAND.

### Screening test battery

**Figure 1:**
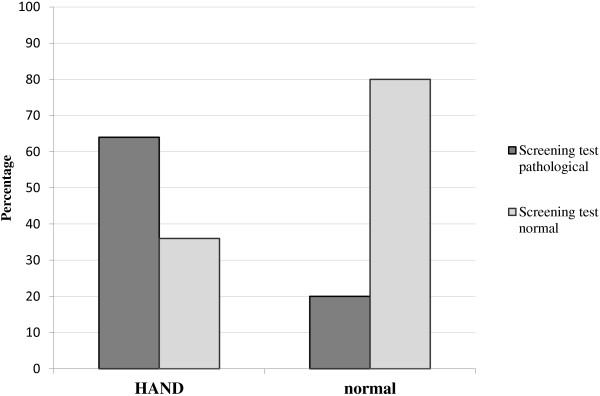
**Performance of the screening battery according to the presence of HAND at the in-depth neuropsychological examination.**

The screening battery revealed neurocognitive deficits in 17 of 30 (57%) patients (Figure 1 and Table 3), corresponding to a sensitivity of 64% (95% confidence interval (CI 42-82%), a specificity of 80% (95% CI 28-99%), a PPV of 94% (95% CI 71-99%) and a NPV of 31% (95% CI 9-61%). Almost all patients with a pathological screening test (16/17, 94%) had a pathological result on their neuropsychological assessment. However, among the 13 patients with a normal screening result, 9 (69%) had HAND at the in-depth neuropsychological examination, i.e. were false negative. If only non-complaining patients (n = 18) were considered, i.e. patients not complaining of memory loss or difficulties in concentrating, the screening battery yielded a sensitivity of 75% (95% CI 48-93%) and a specificity of 100% (95% CI 19-100%), a PPV of 100% (95% CI 73-100%) and a NPV of 33% (95% CI 5-77%). If only patients with memory loss or difficulties to concentrate (n = 12) were considered, the screening battery had a sensitivity of 44% (95% CI 14-79%), specificity of 67% (95% CI 12-94%), a PPV of 80% (95% CI 29-97%) and a NPV of 29% (95% CI 5-71%). If results of the screening test battery were combined with those of the questionnaire (either one or both tests positive), a sensitivity of 84% (95% CI 64-95%), a specificity of 40% (95% CI 6-85%), PPV of 88% (95% CI 68-97%) and NPV of 33% (95% CI 5-77%) could be reached.

**Table 3:** **Performance of the screening battery according to the presence of HAND at the in-depth neuropsychological examination**

		In-depth neuropsychological assessment		
		HAND	Normal cognition	
**Screening result**	positive	16	1	17
		sensitivity = 64%		
		PPV = 94%		
	negative	9	4	13
			specificity = 80%	
			NPV = 31%	
		25	5	30

Comparison of results of the screening battery to those of the in-depth neurolopsychological assessment are shown in Table 3 and Figure 1.

Baseline characteristics of the study population according to results of the screening battery are shown in Table 4. Test results were independent from demographic patients’ characteristics, CD4 cell count, co-medication and cART. The overall duration of the screening test was 25 minutes (IQR 23–29), shorter if the patient had a normal neuropsychological assessment (21 minutes, IQR 20–25).

**Table 4:** **General characteristics of the study population (n = 30) according to results of the screening battery**

	Pathological screening test N = 17		Normal screening test N = 13		p-value
	n	%	n	%	
Median Age, IQR	55	49-64	49	43-61	0.201
Males	15	88	11	85	0.591
Prior AIDS-defining condition	6	35	5	38	0.579
Median CD4 cell count, IQR	565	405-738	695	531-891	0.187
Median years of education, IQR	12	11-13	13	12-13	0.336
cART containing efavirenz	6	35	2	15	0.212
Co-medication with psychotropic drugs (antidepressants, antipsychotics)	5	29	2	15	0.427
Memory loss or difficulties to concentrate reported by the patient*	5	29	7	54	0.164
Depressive symptoms*	2	12	1	8	0.603
Median duration of screening battery, minutes, IQR	25	25-29	23	21-26	0.099

*According to the questionnaire.

### Acceptance of the screening battery

The overall acceptance of the screening battery was excellent. Most patients (83%) estimated the screening test as valuable and not worrisome, and were interested in the results. Most participants (97%) considered the instructions for the test given by the study nurses as clear and the test battery as not difficult or partly difficult in 43% and 57% of patients, respectively. Privacy was not affected by the test according to 93% of patients, and nobody reported that the test was too long. Study nurses also judged the screening battery as not too difficult for patients, valuable and not worrisome, and not too long.

## Discussion

In this study, investigating a specific combination of tests comprising a short questionnaire and a battery of selected neuropsychological tests for mild neurocognitive deficits in 30 HIV-infected individuals receiving cART, we found a moderate sensitivity and specificity when comparing to the in-depth neuropsychological examination serving as the criterion standard. Importantly, we found a high acceptance rate by patients and nurses. The sensitivity and specificity for this screening battery was increased in patients not complaining of memory loss or difficulties in concentration. If we combined results of the screening battery with those from the questionnaire (either one or both tests positive) we reached a sensitivity of 84% with a PPV of 88% and a NPV of 33%, making this screening strategy, administered by nurses, a simple, well accepted tool to screen treated HIV-infected individuals for mild neurocognitive disorders.

The prevalence of HAND in our study population was high (83%). This is in agreement with other studies (Simioni et al. 2010). This is remarkable since all patients were not of older age, had no major psychiatric diseases and were not currently injecting drug users. Also, almost all patients had experienced a long school and professional education. Furthermore, nearly all patients had a suppressed viral load and were immunologically stable under continuous cART.

We consider the two viral load measurements in two patients (58 copies/mL and 65 copies/mL, respectively) as technical blips, however, we cannot rule out a low level viral replication. Five patients had an elevated HIV viral load (range 86–3594 copies/mL) within 6 months before this investigation. The patient with 3594 copies/ml did not take his medication at this time. Within the last 3 months before the examination, however, the viral load was suppressed.

Importantly, we could neither find any association with a cART regimen, in particular with efavirenz-based cART, nor with co-morbidities possibly affecting the neurocognitive performance. Interestingly, objective evidence of HAND was slightly more frequent in patients without complaints suggesting that an easy screening tool is very valuable before neuropsychological examination with more sophisticated instruments. Complaints about memory loss and difficulties to concentrate are difficult to interpret and also frequently reported by HIV-negative individuals (20-70%) without objective cognitive impairment (St John & Montgomery 2002; Reid & Maclullich 2006).

One of the problems encountered with investigating a new screening battery is the lack of a clear criterion standard. However, the in-depth neuropsychological examination has been well validated for cognitive assessment. The search for a good, easy-to-perform screening test is still justified, as the international HDS (Sacktor et al. 2005) and the HDS (Power et al. 1995) that are widely used as screening tests to identify individuals at risk for HAD (Sacktor et al. 2005), are not enough sensitive to detect mild forms of neurocognitive deficits. However, the HIV dementia scale with a cut-off of 14 points was shown to have a sensitivity of 83%, specificity of 63% and a PPV of 92% to detect HAND in patients with complaints and a sensitivity of 88%, specificity of 67% and PPV of 82% in non-complaining patients (Simioni et al. 2010). As a preliminary but encouraging result of this pilot study, our screening battery showed a similar accuracy as the HDS. An advantage may be that our screening battery does not comprise the anti-saccadic eye movement task, which can be challenging for the examiner. In addition to using cognitive tests only it incorporates the information about the patient's subjective cognitive impairment. Importantly, our approach to improve the screening for HAND requires a great deal of further work. In line with the suggestions outlined by Kamminga et al. (2013), at least the following points need to be addressed: (a) use of a representative sample of the HIV population, (b) inclusion of a control (HIV-) group with similar characteristics to optimally assess HAND specificity, (c) a more explicit rationale for screen impairment criteria, (d) reporting of all standard criterion validity indexes, (e) reporting of construct validity, and (f) assessing the longitudinal validity of the screening tool including correction for practice effects. Moreover, acceptability was excellent by patients and nurses.

We acknowledge some limitations: First, the number of patients was too small to draw conclusions regarding specific associations for neurocognitive deficits and cART regimen, e.g. efavirenz-based treatment that may impact on the central nervous system, or specific co-morbidities. Second, we did not perform MRI examinations of the brain nor lumbar punctures to exclude other causes of neurocognitive impairment than HIV. Third, the HIV dementia scale (Sacktor et al. 2005) was not performed in our study population, so that a direct comparison with our screening test was not possible. Forth, the use of the same variable to assess different cognitive domains is problematic. This variable receives an unjustified importance and may lead to invalid results. Furthermore, combining the results of a screening battery with a questionnaire for subjective cognitive impairment is not in line with the Frascati criteria (Antinori et al. 2007). However, this more comprehensive screening approach will allow the medical staff to detect not only patients with HAND, but also others, who might be in need of medical care, which in our view is an advantage.

In our study the prevalence of HAND was high. This may be due to the fact that patients with HCV co-infection, history of ischemic stroke, drug use and previous cerebral opportunistic infections were included in this study.

Drug use was assessed by self-report. We acknowledge self-report and potential interference of drug use as a limitation. Future research should also exclude patients with occasional drug consumption.

This study also has several strengths: First, this is the first study investigating a short screening battery with selected neuropsychological tests that were administered by nurses. Second, all 30 patients were underwent comprehensive neuropsychological assessments. Third, the fact that all patients were participating in the prospective Swiss HIV Cohort Study enabled us to assess important co-morbidities such as depression or syphilis and the corresponding treatments.

## Conclusion

In conclusion, our study demonstrates that screening for neurocognitive deficits is likely to identify milder forms of cognitive disorders even in non-complaining patients. A short questionnaire combined with a small battery of selected neuropsychological tests is a short, easy-to-perform screening tool for HIV-infected individuals.

## References

[CR1] Antinori A, Arendt G, Becker JT, Brew BJ, Byrd DA, Cherner M, Clifford DB, Cinque P, Epstein LG, Goodkin K, Gisslen M, Grant I, Heaton RK, Joseph J, Marder K, Marra CM, McArthur JC, Nunn M, Price RW, Pulliam L, Robertson KR, Sacktor N, Valcour V, Wojna VE (2007). Updated research nosology for HIV-associated neurocognitive disorders. Neurology.

[CR2] Aster M, Neubauer A, Horn R (2006). Wechsler Intelligenztest für Erwachsene (WIE). Manual.

[CR3] Benedict RHB, Schretlen D, Groninger L, Brandt J (1998). The Hopkins verbal learning test-revised: normative data and analysis of inter-form and test-retest reliability. Clinical Neuropsychology.

[CR4] Berghuis JP, Uldall KK, Lalonde B (1999). Validity of two scales in identifying HIV-associated dementia. Journal of Acquired Immune Deficiency Syndromes.

[CR5] Calabrese P, Kalbe E, Kessler J, Calabrese P, Kalbe E, Kessler J (2004). Ein neuropsychologisches screening zur Erfassung kognitiver Störungen bei MS-Patienten – Das Multiple Sklerose Inventarium Cognition (MUSIC). NeuroPsychol.

[CR6] Carey CL, Woods SP, Rippeth JD, Gonzalez R, Moore DJ, Marcotte TD, Grant I, Heaton RK, HNRC Group (2004). Initial validation of a screening battery for the detection of HIV-associated cognitive impairment. Clinical Neuropsychology.

[CR7] Carter SL, Rourke SB, Murji S, Shore D, Rourke BP (2003). Cognitive complaints, depression, medical symptoms, and their association with neuropsychological functioning in HIV infection: a structural equation model analysis. Neuropsychology.

[CR8] Cysique LA, Brew BJ (2009). Neuropsychological functioning and antiretroviral treatment in HIV/AIDS: a review. Neuropsychology Review.

[CR9] D’Elia LF, Satz P, Uchiyama CL, White T (1996). Color Trails Test. Professional manual.

[CR10] Delis DC, Kramer JH, Kaplan E, Ober BA (1987). The California Verbal Learning Test.

[CR11] Egger M, May M, Chêne G, Phillips AN, Ledergerber B, Dabis F, Costagliola D, D'Arminio Monforte A, de Wolf F, Reiss P, Lundgren JD, Justice AC, Staszewski S, Leport C, Hogg RS, Sabin CA, Gill MJ, Salzberger B, Sterne JA (2002). Prognosis of HIV-1-infected patients starting highly active antiretroviral therapy: a collaborative analysis of prospective studies. Lancet.

[CR12] Fimm B, Zimmermann P (2009). Test zur Aufmerksamkeitsprüfung (TAP) Version 2.2.

[CR13] Gonzalez R, Vassileva J, Bechara A, Grbesic S, Sworowski L, Novak RM, Nunnally G, Martin EM (2005). The influence of executive functions, sensation seeking, and HIV serostatus on the risky sexual practices of substance-dependent individuals. Journal of the International Neuropsychological Society.

[CR14] Härting C, Markowitsch HJ, Neufeld H, Calabrese P, Deisinger K, Kessler J (2000). Deutsche Adaption der revidierten Fassung der Wechsler Memory Scale (WMS- R).

[CR15] Helmstadter C, Lendt M, Lux S (2001). Verbaler Lern- und Merkfähigkeitstest: VLMT, Manual.

[CR16] Hinkin CH, Castellon SA, Durvasula RS, Hardy DJ, Lam MN, Mason KI, Thrasher D, Goetz MB, Stefaniak M (2002). Medication adherence among HIV + adults: effects of cognitive dysfunction and regimen complexity. Neurology.

[CR17] Jaggy C, von Overbeck J, Ledergerber B, Schwarz C, Egger M, Rickenbach M, Furrer HJ, Telenti A, Battegay M, Flepp M, Vernazza P, Bernasconi E, Hirschel B (2003). Mortality in the Swiss HIV Cohort Study (SHCS) and the Swiss general population. Lancet.

[CR18] Jones BN, Teng EL, Folstein MF, Harrison KS (1993). A new bedside test of cognition for patients with HIV infection. Annals of Internal Medicine.

[CR19] Joska JA, Gouse H, Paul RH, Stein DJ, Flisher AJ (2010). Does highly active antiretroviral therapy improve neurocognitive function? A systematic review. Journal of Neurovirology.

[CR20] Kamminga J, Cysique LA, Lu G, Batchelor J, Brew BJ (2013). Validity of cognitive screens for HIV-associated neurocognitive disorder: a systematic review and an informed screen selection guide. Current HIV/AIDS Reports.

[CR21] Marder K, Albert SM, McDermott MP, McArthur JC, Schifitto G, Selnes OA, Sacktor N, Stern Y, Palumbo D, Kieburtz K, Cohen B, Orme C, Epstein LG (2003). Inter-rater reliability of a clinical staging of HIV-associated cognitive impairment. Neurology.

[CR22] Mocroft A, Ledergerber B, Katlama C, Kirk O, Reiss P, d'Arminio Monforte A, Knysz B, Dietrich M, Phillips AN, Lundgren JD (2003). Decline in the AIDS and death rates in the EuroSIDA study: an observational study. Lancet.

[CR23] Morris JC, Edland S, Clark C, Galasko D, Koss E, Mohs R, van Belle G, Fillenbaum G, Heyman A (1989). The Consortium to Establish a Registry for Alzheimer’s Disease (CERAD). Part I. Clinical and neuropsychological assessment of Alzheimer’s disease. Neurology.

[CR24] Nelson HE (1976). A modified card sorting test sensitive to frontal lobe defects. Cortex.

[CR25] Power C, Selnes OA, Grim JA, McArthur JC (1995). HIV Dementia Scale: a rapid screening test. Journal of Acquired Immune Deficiency Syndromes and Human Retrovirology.

[CR26] Regard M, Strauss E, Knapp P (1982). Children’s production on verbal and non-verbal fluency tasks. Perceptual and Motor Skills.

[CR27] Reid LM, Maclullich AMJ (2006). Subjective memory complaints and cognitive impairment in older people. Dementia and Geriatric Cognitive Disorders.

[CR28] Rey A (1941). L’examen psychologique dans les cas d’encephalopathie traumatique. Archives of Psychology.

[CR29] Robertson KR, Smurzynski M, Parsons TD, Wu K, Bosch RJ, Wu J, McArthur JC, Collier AC, Evans SR, Ellis RJ (2007). The prevalence and incidence of neurocognitive impairment in the HAART era. AIDS.

[CR30] Robertson K, Liner J, Heaton R (2009). Neuropsychological assessment of HIV-infected populations in international settings. Neuropsychology Review.

[CR31] Rourke SB, Halman MH, Bassel C (1999). Neurocognitive complaints in HIV-infection and their relationship to depressive symptoms and neuropsychological functioning. Journal of Clinical and Experimental Neuropsychology.

[CR32] Ruff RM, Parker SB (1993). Gender- and age-specific changes in motor speed and eye-hand coordination in adults: normative values for the Finger Tapping and Grooved Pegboard Tests. Perceptual and Motor Skills.

[CR33] Sacktor NC, Wong M, Nakasujja N, Skolasky RL, Selnes OA, Musisi S, Robertson K, McArthur JC, Ronald A, Katabira E (2005). The International HIV Dementia Scale: a new rapid screening test for HIV dementia. AIDS.

[CR34] Simioni S, Cavassini M, Annoni JM, Rimbault Abraham A, Bourquin I, Schiffer V, Calmy A, Chave JP, Giacobini E, Hirschel B, Du Pasquier RA (2010). Cognitive dysfunction in HIV patients despite long-standing suppression of viremia. AIDS.

[CR35] Singh D, Joska JA, Goodkin K, Lopez E, Myer L, Paul RH, John S, Sunpath H (2010). Normative scores for a brief neuropsychological battery for the detection of HIV-associated neurocognitive disorder (HAND) among South Africans. BMC Research Notes.

[CR36] Skinner S, Adewale AJ, DeBlock L, Gill MJ, Power C (2009). Neurocognitive screening tools in HIV/AIDS: comparative performance among patients exposed to antiretroviral therapy. HIV Medicine.

[CR37] St John P, Montgomery P (2002). Are cognitively intact seniors with subjective memory loss more likely to develop dementia?. International Journal of Geriatric Psychiatry.

[CR38] Stöckle M, Elzi L, Rockstroh JK, Battegay M (2012). Morbidity and mortality in HIV infection. Internist (Berl).

[CR39] Stroop JR (1935). Studies of interference in serial verbal reactions. Journal of Experimental Psychology.

[CR40] The Antiretroviral Therapy Cohort Collaboration (2008). Life expectancy of individuals on combination antiretroviral therapy in high-income countries: a collaborative analysis of 14 cohort studies. Lancet.

[CR41] Tombaugh TN, Rees L, McIntyre N (1998). Normative data for the Trail Making Test. Personal communication cited in Spreen and Strauss. A compendium of neuropsychological tests: Administration, norms and commentary (2nd ed.).

[CR42] Tozzi V, Balestra P, Bellagamba R, Corpolongo A, Salvatori MF, Visco-Comandini U, Vlassi C, Giulianelli M, Galgani S, Antinori A, Narciso P (2007). Persistence of neuropsychologic deficits despite long-term highly active antiretroviral therapy in patients with HIV-related neurocognitive impairment: prevalence and risk factors. Journal of Acquired Immune Deficiency Syndromes.

[CR43] Trail Making Test (1944). Army Individual Test Battery. Manual of directions and scoring.

[CR44] Trull TJ, Vergés A, Wood PK, Jahng S, Sher KJ (2012). The structure of Diagnostic and Statistical Manual of Mental Disorders (4th edition, text revision) personality disorder symptoms in a large national sample. Personal Disorder.

[CR45] Vance DE, Struzick TC (2007). Addressing risk factors of cognitive impairment in adults aging with HIV: a social work model. Journal of Gerontological Social Work.

[CR46] Weber R, Ruppik M, Rickenbach M, Spoerri A, Furrer H, Battegay M, Cavassini M, Calmy A, Bernasconi E, Schmid P, Flepp M, Kowalska J, Ledergerber B (2012). Decreasing mortality and changing patterns of causes of death in the Swiss HIV Cohort Study. HIV Medicine.

[CR47] Woods SP, Moore DJ, Weber E, Grant I (2009). Cognitive neuropsychology of HIV-associated neurocognitive disorders. Neuropsychology Review.

[CR48] The pre-publication history for this paper can be accessed here:http://www.biomedcentral.com/2050-7283/2/21/prepub

